# Enhancing energy absorption through sequential instabilities in mechanical metamaterials

**DOI:** 10.1098/rsos.230762

**Published:** 2023-08-30

**Authors:** Adam Bekele, M. Ahmer Wadee, Andrew T. M. Phillips

**Affiliations:** Department of Civil and Environmental Engineering, Imperial College London, London SW7 2AZ, UK

**Keywords:** mechanical metamaterials, structural instability, mathematical modelling, finite-element analysis, physical experiments

## Abstract

Structural components designed to absorb energy and shield a more valuable structure ideally require mechanical properties that combine a relatively high load-carrying capacity followed by a practically zero stiffness. This ensures that a specified energy quantity may be absorbed within a limited displacement and that any stress transfer to the valuable structure is minimized. Material damage has been historically mobilized to provide such properties, but this obviously renders such components to be single-use. By contrast, mobilization of elastic instability can also provide the desired combination of properties but without necessarily damaging the material. This reveals an intriguing possibility of such components being potentially repairable and theoretically re-usable with no significant loss in performance. A series of analytical, finite-element and experimental studies are presented for a bespoke mechanical metamaterial arrangement that is designed to buckle sequentially and behave with the desired ‘high strength–low stiffness’ characteristic. It is found that the various axial and rotational stiffnesses associated with the geometric arrangement and its constituent connections may be tuned to provide the desired mechanical behaviour within the elastic range and delay the onset of significant damage, thereby rendering the concept of harnessing instability to be feasible.

## 1. Introduction

Structural instability, or buckling, is generally regarded as a phenomenon that is best avoided with design processes ensuring its prevention. However, it has also been known for a relatively long time now that the change in stiffness arising from the buckling process can be harnessed for advantageous purposes [1–4]. Indeed, more recent advances have seen the harnessing of buckling in a wide range of applications, including vibration isolation, shape adaptation and energy absorption [5,6]. In the field of structural engineering, the naturally stable post-buckling of plates has been exploited since the 1940s owing to the pioneering work of, amongst others, Winter [7]. More recently, particularly in the fields of mechanical and aeronautical structures, the actual mechanical process of buckling has been harnessed in so-called smart shape-morphing materials and structures that are designed to switch from one geometric form to another under particular loading ranges [8], which also has significance in the field of energy harvesting [9]. In the design of mechanical metamaterials, which have bespoke mechanical properties owing to their geometry, the nonlinear behaviour of their internal structure can result in potentially exploitable features for both energy- and motion-related applications [10]—this is especially the case for auxetic materials that are designed to have a negative Poisson’s ratio [11–17].

**Figure 1.**
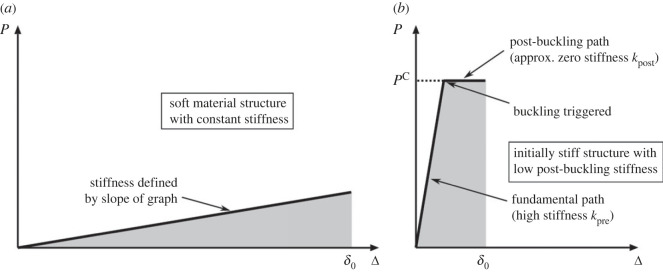
Idealized mechanical response of an (*a*) soft and (*b*) stiff elastic structure that buckles showing the applied load *P* versus displacement Δ where the shaded regions represent the same total strain energy absorbed in each structure. In (*b*), the gradients of the function *P*(Δ) labelled *k*_pre_ and *k*_post_ represent the fundamental and post-buckling stiffnesses, respectively, while *P*^C^ represents the critical buckling load. Note that in (*b*), in contrast with (*a*), the required energy can be absorbed with a much smaller displacement, denoted by *δ*_0_, which reduces the necessary size of the energy absorption system.

Recent world events have brought into sharp focus the need for certain critically important buildings and infrastructure to have protective layers shielding from impact or blast. Therefore, the quest for efficient energy absorbing systems has obtained crucial importance. Although soft materials with a relatively high yield stress would technically be able to perform this task, examples of which are polymeric composites and foam-filled thin-walled structures [18,19], they require a significant volume or thickness of material to accommodate the large deformation required to absorb the required energy quantity; see figure 1*a*, where *δ*_0_ represents the necessary deformability for such a system to be effective. However, cellular structures with bespoke geometrical configurations can be designed with a high load-carrying capacity and minimal post-buckling stiffness [20,21]. This has the potential to achieve the same energy absorbency but for a fraction of the compression displacement *δ*_0_, as represented in figure 1*b*. This can be achieved through material viscoelasticity or through the loss of stiffness due to plasticity [22]. However, the same can also be achieved through using elastic snap-through instabilities [23,24], where deformations can be reversed without necessarily damaging the material; this requires a given geometry to jump from one configuration to another when an applied load reaches a critical level [25]. The desired mechanical response, shown in figure 1*b*, has an initially high stiffness and large load-carrying capacity, followed by a quasi-zero post-buckling stiffness. While the latter (stable) low-stiffness post-buckling response restricts a transfer of large impact forces to the protected structure, the high initial stiffness *k*_pre_ and high load-carrying capacity maximize the energy absorbency of the system, measured by evaluating the area under the load–displacement curve, without needing a large axial displacement, as would be required for the aforementioned structures made from soft materials. Shan *et al.* [26] have designed multistable metamaterials with tilted beam arrangements that can lock in strain energy during its geometrical ‘phase transition’, which they refer to as ‘energy trapping’. Similarly, Che *et al.* [27] proposed multistable metamaterials with the addition of small variations in the cell geometry to obtain a deterministic deformation sequence that is more predictable than a lattice with identical repeating unit cells.

Of course, lots of experimental and numerical work already exists within the literature on investigating different kinds of metamaterials for enhancing energy absorption and structural isolation performance of protective cellular structures [28,29], including those investigating two- and three-dimensional cellular structures [11,22,30] and the aforementioned work on cells designed to snap-through without inherently damaging the lattice material [23,24]. By contrast, the principal contribution of the present work is to devise a mechanics-based analytical methodology that complements the existing work by using rigid links and springs to model the behavioural transitions at the cellular level. The aim of such an approach is to augment the experimental and numerical approaches such that subtle changes in the cellular arrangement can be understood at a more fundamental level. A bespoke bistable cellular configuration is devised and analysed at the unit and multiple cell levels to capture the influence of the specific geometry, alongside the variation of rotational and axial stiffnesses, on the overall nonlinear mechanical response using minimum total potential energy principles. The findings are subsequently adapted to more physically realistic finite-element models, which facilitates the analysis of lattices at much larger scales. Finally, to verify the numerical model and demonstrate the feasibility of such parametric changes in practice, physical experiments are conducted on 3D-printed specimens. Following a discussion on the implications, conclusions are drawn.

## 2. Analytical modelling

**Figure 2.**
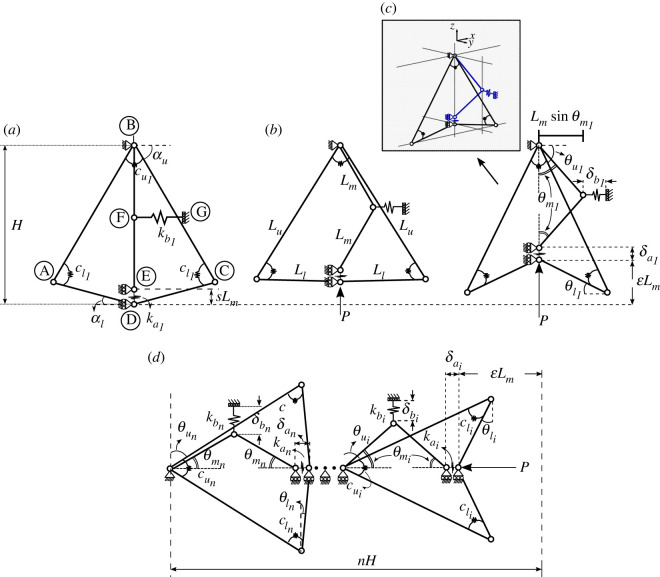
Schematic of the flint arrowhead configuration highlighting all of the relevant rotation angles, spring stiffnesses and lengths; (*a*–*c*), respectively, represent the unit cell at three stages of axial compression, while (*d*) represents a stack of *n* cells, where *i* = {1, 2, …, *n*}. Note that the spring FG acts effectively out of the plane of the rest of the cell.

A unit cell configuration, referred to presently as a ‘flint arrowhead’ and shown in figure 2, is selected owing to its increased shear resistance and uni-directional deformation mechanism. This bi-stable geometry can snap through from the convex flint arrowhead geometry (see figure 2*a* for the unloaded flint configuration), whereby the in-plane cellular structure initially expands laterally under the compressive load *P* (figure 2*b*), to an auxetic arrowhead [31] (figure 2*c*), which has an effective negative Poisson’s ratio owing to its re-entrant angles and contracts laterally under further compressive displacement $EL_{m}$. A mechanical model of this cellular configuration comprising rigid links and springs is initially analysed to obtain a deeper understanding of the geometrically nonlinear response. This two-dimensional unit cell has an in-plane arrowhead, whereas the middle strut can buckle out of plane as shown by the small shaded isometric drawing within figure 2. To determine the influence of the axial and bending stiffness of the middle strut, two longitudinal springs joining the nodes at D and E alongside F and G with spring stiffness values $k_{a_{1}}$ and $k_{b_{1}}$, respectively, are introduced. Rotational springs are provided for the in-plane arrowhead, at nodes A, B and C, with congruent stiffness values $c_{l_{1}}$ for the lower springs at nodes A and C, while a stiffness of $c_{u_{1}}$ is provided for the upper rotational spring at node B. The outer rigid links have lengths *L*_*u*_ and *L*_*l*_ for the upper and lower members, respectively, while the two rigid links within the middle strut have equal lengths of *L*_*m*_. The original length DE of the lower longitudinal spring is given as a factor of the middle strut length *sL*_*m*_. The initial angles of inclination for the lower and upper links of the arrowhead are *α*_*l*_ and *α*_*u*_, respectively, with specific values of these given in table 1. When the axial load *P* is applied, the changing angles of inclination to the horizontal are $\theta_{l_{1}}$ and $\theta_{u_{1}}$ for the lower and upper links, as shown in figure 2*c*. However, the angle to the vertical created by the buckling of the middle strut links is given as $\theta_{m_{1}}$. Finally, the displacements of the longitudinal springs, which arise from the changing lengths of DE and FG, respectively, as the unit cell compresses are given by $\delta_{a_{1}}$ and $\delta_{b_{1}}$, while the overall end-shortening for the loading is given as $EL_{m}$.

**Table 1.** Initial dimensions of the flint unit cell with lower and upper inclination angles *α*_*l*_ and *α*_*u*_, respectively, ratio of lower spring *s* and lower member length *L*_*l*_. As shown by the formulations in §2.1, the other terms can be deduced from these dimensions.

*α*_*l*_ (deg)	*α*_*u*_ (deg)	*s*	*L*_*l*_ (mm)
$15^{\circ }$	$60^{\circ }$	0.2	3.5

Figure 2*d* shows a series of cells referred to as a ‘stack’ of flint arrowhead cells. The lengths of the rigid links are all equivalent to those of the unit cell and the only differences are in the subscripts *i*, where *i* = {1, 2, …, *n*}, which indicate the position of each cell. The overall length of the stack in the unloaded state, required for axial end-shortening calculations, is given by *nH*, where *n* is the total number of cells in the stack.

### 2.1. Potential energy formulation

The equilibrium equations for the flint arrowhead mechanical model, as shown in figure 2, can be derived from the principle of stationary total potential energy [25] for a system with *n* cells lying in series. The total potential energy *V* is defined thus:

2.1 V=U−PΔ.

Here, the first term *U* is the total strain energy stored in all the springs, while the second term is the work done by the external load *P* with Δ being the distance the external load moves in the loading direction. The total strain energy *U* comprises the sum of the contributions from both the longitudinal and rotational springs, *U*_*L*_ and *U*_*R*_, respectively, where *U*_*L*_ is given thus

2.2 $$U_{L}=\frac{L_{m}^{2}}{2}\sum_{i=1}^{n}[k_{a_{i}}\Delta_{a_{i}}^{2}+k_{b_{i}}\sin^{2}\theta_{m_{i}}],$$

where *i* is an index representing a particular cell such that *i* = {1, 2, …, *n*} with $\Delta_{a_{i}}$ being the normalized change in length of the longitudinal spring of stiffness $k_{a_{i}}$, written in terms of *s*, *L*_*m*_ and $\delta_{a_{i}}$ (cf. figure 2*a* and 2*c*), thus:

2.3 $$\Delta_{a_{i}}L_{m}=sL_{m}-\delta_{a_{i}},$$

with $\Delta_{a_{i}}$ being expressed in terms of $\theta_{m_{i}}$ and $\theta_{l_{i}}$ such that:

2.4 $$\Delta_{a_{i}}=s+2\cos\theta_{m_{i}}-\frac{L_{l}\sin\theta_{l_{i}}+L_{u}\sin\theta_{u_{i}}}{L_{m}}.$$

The length terms can also be simplified such that the constant lengths *L*_*u*_ and *L*_*m*_ can be written in terms of length *L*_*l*_ alongside the initial upper and lower angles *α*_*u*_ and *α*_*l*_, respectively,

2.5 $$L_{u}=L_{l}\frac{\cos\alpha_{l}}{\cos\alpha_{u}}\;\mathrm{and}\;L_{m}=\frac{L_{l}}{\left(s+2\right)}(\cos\alpha_{l}\tan\alpha_{u}+\sin\alpha_{l}).$$

Furthermore, $\theta_{u_{i}}$ can be written in terms of $\theta_{l_{i}}$

2.6 $$\theta_{u_{i}}=\text{arccos}\left(\frac{\cos\alpha_{u}\cos\theta_{l_{i}}}{\cos\alpha_{l}}\right).$$

Using the same principles, the strain energy stored in the rotational springs *U*_*R*_ is given by:

2.7 $$U_{R}=\frac{1}{2}\sum_{i=1}^{n}[c_{l_{i}}{\left(\alpha_{u}+\alpha_{l}-\theta_{u_{i}}-\theta_{l_{i}}\right)}^{2}+c_{u_{i}}{\left(\theta_{u_{i}}-\alpha_{u}\right)}^{2}],$$

while the total end-shortening $\Delta =EL_{m}$, which is given by the expression

2.8 $$EL_{m}=L_{m}\sum_{i=1}^{n}[\Delta_{a_{i}}-2\cos\theta_{m_{i}}]-2nL_{m}.$$

Given the constituent terms established, the total potential energy *V* can be formulated. Invoking the principle of stationary *V* with respect to the generalized coordinates $\theta_{l_{i}}$ and $\theta_{m_{i}}$ provides the governing nonlinear equilibrium equations, i.e. $\partial V/\partial \theta_{m_{i}}=0$ and $\partial V/\partial \theta_{l_{i}}=0$, which are solved within the well-known numerical continuation and bifurcation software AUTO-07P [32]. The following non-dimensional terms are used in presenting the results that follow; these are obtained simply by dividing *V* through by $k_{a_{1}}L_{m}^{2}$

2.9 $${\tilde{k}}_{a_{i}}=\frac{k_{a_{i}}}{k_{a_{1}}},\;{\tilde{k}}_{b_{i}}=\frac{k_{b_{i}}}{k_{a_{1}}},\;{\tilde{c}}_{l_{i}}=\frac{c_{l_{i}}}{k_{a_{1}}L_{m}^{2}},\;{\tilde{c}}_{u_{i}}=\frac{c_{u_{i}}}{k_{a_{1}}L_{m}^{2}},\;\tilde{P}=\frac{P}{k_{a_{1}}L_{m}}.$$

### 2.2. Results

#### 2.2.1. Unit cell behaviour

**Figure 3.**
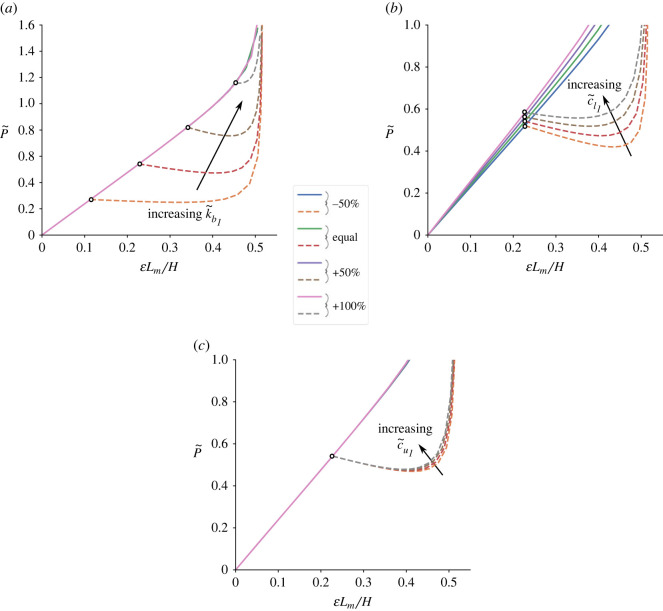
Normalized load $\tilde{P}$ versus normalized axial end-shortening $EL_{m}/H$ responses of a flint arrowhead unit cell with respective variations to the normalized spring stiffnesses of (*a*) the middle strut ${\tilde{k}}_{b_{1}}$, (*b*) the in-plane lower springs ${\tilde{c}}_{l_{1}}$ and (*c*) the in-plane upper spring ${\tilde{c}}_{u_{1}}$. In each case, the normalized spring stiffnesses are either kept equal to each other or the respectively labelled stiffness is reduced by 50%, increased by 50%, or increased by 100%.

Figure 3 shows a series of normalized load $\tilde{P}$ versus normalized end-shortening $EL_{m}/H$ equilibrium paths for a flint arrowhead unit cell where modifications are made to the out-of-plane ${\tilde{k}}_{b_{1}}$, and in-plane ${\tilde{c}}_{l_{1}}$ and ${\tilde{c}}_{u_{1}}$ spring stiffnesses. In the three graphs that are plotted, cases where all the non-dimensional spring stiffnesses are equal are presented alongside cases where the specified normalized stiffness value is either increased or decreased by 50%, or that value is increased by 100%. To maintain equal spring stiffness initially, ${\tilde{k}}_{b_{1}}$ takes a unit value, whereas parameters ${\tilde{c}}_{l_{1}}$ and ${\tilde{c}}_{u_{1}}$ take a value of 0.106, owing to the $L_{m}^{2}$ term in their normalization, as seen in equation (2.9). In figure 3*a*, the middle strut longitudinal normalized spring stiffness ${\tilde{k}}_{b_{1}}$ is varied; the initial gradient, representing the fundamental axial stiffness of the unit cell under compression, is seen to be independent of ${\tilde{k}}_{b_{1}}$. However, it is evident that an increase to ${\tilde{k}}_{b_{1}}$ leads to a significant increase in the buckling load since it restricts the out-of-plane buckling of the middle strut. The post-buckling response is only slightly affected by these modifications, with the lowest curve showing the best plateauing post-buckling response, necessary for structural isolation applications (figure 1); higher ${\tilde{k}}_{b_{1}}$ values with the higher dashed lines bifurcating from the fundamental path introduce more unstable post-buckling and a smaller end-shortening range before restabilization from the individual cell folding up occurs, effectively triggering densification, whose paths all appear to converge onto the same normalized end-shortening value of approximately 50%. If ${\tilde{k}}_{b_{1}}$ is excessively large, the middle strut does not buckle and the cell transitions from conventional to auxetic behaviour without a plateau; an undesirable result in the present context.

Figure 3*b* shows the lower in-plane rotational spring stiffness ${\tilde{c}}_{l_{1}}$ being varied, with the fundamental stiffness and the load-carrying capacity increasing as ${\tilde{c}}_{l_{1}}$ is increased. However, the effects are less prominent compared with those observed in figure 3*a*. The post-buckling stiffness transitions from a clearly negative value to the more favoured quasi-zero stiffness response as ${\tilde{c}}_{l_{1}}$ is increased. However, it is worth noting that the plateauing response is curtailed with an early onset of densification as ${\tilde{c}}_{l_{1}}$ is further increased. Figure 3*c* presents the equivalent results while varying the upper rotational spring stiffness ${\tilde{c}}_{u_{1}}$, which appears mainly to affect the latter densification region of the paths. Although the changes are less significant compared with ${\tilde{k}}_{b_{1}}$ and ${\tilde{c}}_{l_{1}}$, higher ${\tilde{c}}_{u_{1}}$ terms produce an earlier onset of densification, which makes intuitive sense since the upper spring rotates significantly mainly after the geometrical phase transition from the conventional ‘flint’ to the ‘auxetic’ arrowhead cellular form. Unlike the other springs that affect important regions in the equilibrium paths, this parameter is less significant since measures should be taken to maximize energy absorption and improve structural isolation performance prior to the mechanical phase where the effect of ${\tilde{c}}_{u_{1}}$ gains prominence. Nevertheless, this rotational spring can still provide a scope for improving the overall response, in combination with the other findings, principally to delay the onset of densification.

#### 2.2.2. Multiple cell stack behaviour

The behaviour of five flint arrowhead cells in series is examined primarily to understand the influence of joining cells together on the overall mechanical response of this cellular architecture. Since the joint stiffnesses dictate the fundamental and post-buckling response, as shown in the unit cell case in §2.2.1, these normalized parameters can be selected to provide the desired set of outcomes. Firstly, the normalized axial stiffness parameters are set to be equal, i.e. ${\tilde{k}}_{a_{i}}=1$ for *i* = {1, 2, 3, 4, 5}, since axial stiffness variations across the stack do not affect the behaviour significantly. Similarly, since the ${\tilde{c}}_{u_{i}}$ parameters were shown to play a minimal role in the load–displacement response, a low value is assigned with equal magnitude for all cells, essentially to delay the densification phase as far as possible. However, the parameters ${\tilde{k}}_{b_{i}}$ showing the relationship between the out-of-plane and in-plane longitudinal spring stiffnesses have been modified within the stack of cells, starting from a low value, where ${\tilde{k}}_{b_{1}}=0.3$, and followed by a 30% increase for consecutive cells, as shown in table 2. Again, since ${\tilde{c}}_{l_{i}}$ is the other significant parameter that affects both the fundamental and post-buckling response, they have been sequentially increased. For the first cell, ${\tilde{c}}_{l_{1}}$ was chosen to be sufficiently low such that it gives a steeper initial gradient and a quasi-zero post-buckling stiffness, but it is increased by 30% for each subsequent cell similar to ${\tilde{k}}_{b_{i}}$. The choice of a 30% step-wise change in both the ${\tilde{k}}_{b_{i}}$ and ${\tilde{c}}_{l_{i}}$ parameters was purely to avoid some convergence issues that were encountered with smaller increases, which prevented the desired full sequential buckling response from being observed in the present numerical study; hence, factors other than the 30% would be completely valid also.

**Table 2.** Normalized stiffness values assigned for the stack of five flint arrowhead cells *i* = {1, 2, 3, 4, 5}, whereby ${\tilde{k}}_{b_{i}}$ is for the out-of-plane middle strut spring, ${\tilde{c}}_{l_{i}}$ is for the lower in-plane springs, while the upper in-plane spring stiffnesses ${\tilde{c}}_{u_{i}}$ and axial spring ${\tilde{k}}_{a_{i}}$ are kept constant at a value of 0.106 and 1, respectively.

*i*	${\tilde{k}}_{b_{i}}$	${\tilde{c}}_{l_{i}}$
1	0.3	0.15
2	0.39	0.195
3	0.507	0.254
4	0.659	0.330
5	0.857	0.428

**Figure 4.**
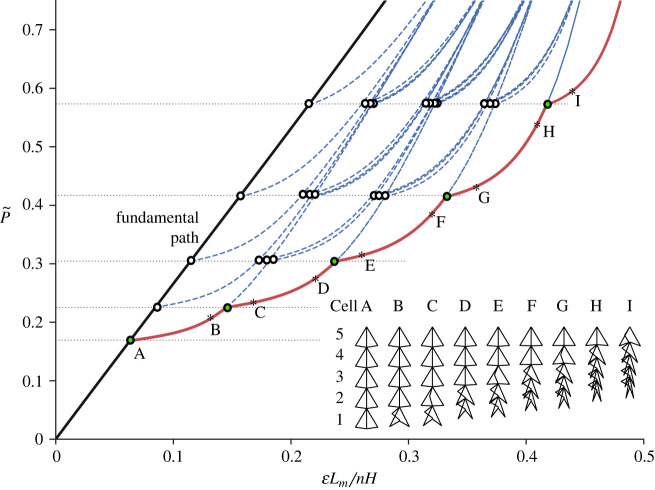
Normalized load $\tilde{P}$ versus normalized axial end-shortening $EL_{m}/H$ for a stack of five flint arrowhead cells. The branches emerging from the bifurcations show all the possible buckling outcomes for each unit cell within the loaded stack. Stack diagrams labelled ‘A’–‘I’ show the cellular deformations on the lowest, minimum energy, equilibrium path.

Figure 4 shows the normalized load $\tilde{P}$ versus normalized axial end-shortening $EL_{m}/nH$ for the five-cell model under axial compression. Owing to the arrangement, where all the cells have different stiffnesses, there are five separate primary bifurcating paths branching from the fundamental path. The first branch relates to the buckling of the least stiff cell, which has the lowest buckling load (cell 1). Similarly, there are four further bifurcation points on the fundamental path signifying non-critical buckling modes. The branches that emerge from the lowest post-buckling path indicate the subsequent buckling of the next most flexible cells. For instance, the bottom set of five branches shown in red, represent the buckling of cell 1, followed by the cells 2–5 in sequence. In practice, the non-critical modes whose paths are shown with blue dot-dashed lines could only feasibly be triggered if the load were to be applied dynamically beyond the critical buckling load through, for instance, an impact or an explosion. Therefore, the paths highlighted in red are practically followed under (quasi-)static loading. To provide a better insight into the mechanics of individual cells within the stack as the sequential buckling occurs, figure 4 also shows cellular deformations with corresponding tags on the branches. The green circles highlight the instant when branching occurs while the asterisks labelled ‘B’–‘I’ show deformations immediately before and after those subsequent bifurcations. The significant differences in stiffness between adjacent cells account for the steady ramping-up of the load with increasing end-shortening in the mechanical response. For smaller stiffness differences, numerical convergence issues ensured that only a subset of the branches were detected but a smaller increase in the load during the sequential buckling response was observed to be practically feasible. The deformed figures ‘A’–‘I’, however, show how each cell buckles independently before the subsequent cell buckles. This perhaps exaggerates the more gradual and intertwined response expected and desired in practice. The increase in buckling load between adjoining cells, as highlighted by the difference in height between the dashed lines in figure 4, could be attributed to the densification of cells that are buckling or have already buckled, which gradually increases the overall structural stiffness.

**Figure 5.**
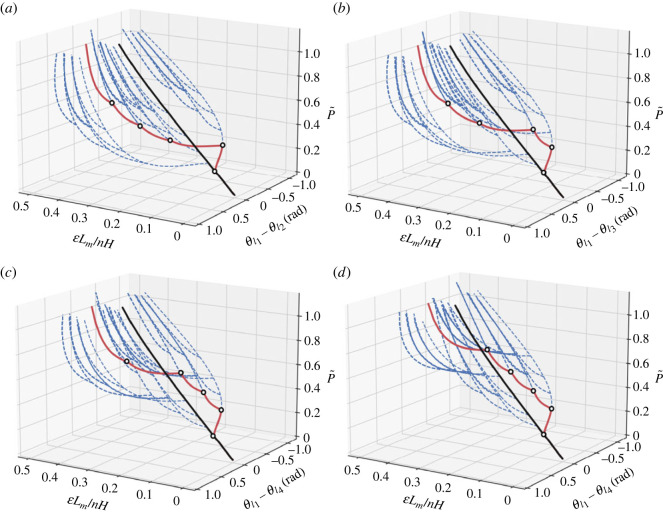
Equilibrium diagrams for the stack of five flint arrowhead cells where the normalized load $\tilde{P}$ and the normalized axial end-shortening $EL_{m}/nH$ versus changes in angles (*a*) $\theta_{l_{1}}-\theta_{l_{2}}$, (*b*) $\theta_{l_{1}}-\theta_{l_{3}}$, (*c*) $\theta_{l_{1}}-\theta_{l_{4}}$ and (*d*) $\theta_{l_{1}}-\theta_{l_{5}}$ are given. Branches highlighted in red correspond to those presented in figure 4.

Figure 5 shows four plots of the normalized load $\tilde{P}$ and the normalized axial end-shortening $EL_{m}/nH$ versus changes in rotation of the lower inclination angles. These graphs provide a more detailed perspective of the sequential buckling response but through changes in the lower angle rotations of each cell. The branches of interest that correspond to the lower set of branches in figure 4 are highlighted in red. Since this response shows a change in angle, the branches trend in the same direction until snap-through is triggered in the cell corresponding to the angle that is compared, which changes the direction of the branching path. For instance, initially in figure 5*a*, since only cell 1 is triggered, only that branch grows in the negative direction until the first bifurcation where the other branches begin to appear. Similarly, when comparing cells 1 and 3, in figure 5*b*, the first and second red branches trend in one direction until the second bifurcation point when the remaining branches start trending in the opposite direction. Similar behaviour is observed throughout the different plots and clearly shows the sequential response of different cells within the stack.

## 3. Finite-element modelling

A dynamic implicit finite-element (FE) method with a quasi-static type loading application is used to analyse all the numerical models presently with two reasons driving this choice. Firstly, dynamic analysis is much more suitable for modelling the snap-through behaviour which often causes the system to flip between static and dynamic responses for various load and displacement combinations as the cell structure jumps from one stable form to another. Secondly, since the system is being considered for energy absorption applications, dynamic FE is much more appropriate for modelling phenomena such as impact or blast, although the accelerations would be significantly higher for such cases and those particular considerations are left for future work. Dynamic analysis features in the commercial FE software ABAQUS [33] whereby minimal inertia effects are introduced to diminish numerical instabilities and improve convergence for determining essentially quasi-static solutions. As indicated above, the snap-through response expected when the flint arrowhead jumps from the conventional flint to the auxetic arrowhead configuration warrants the dynamic analysis, but it also reduces the potential for large stress concentrations within lattices owing to the higher nodal connectivity at the joints. To resolve convergence issues that may occur as a result of these high stress concentrations, some numerical damping is introduced to ensure the solution process remains smooth. The velocity selected in the present analyses is $0.1\;\mathrm{mm}\;s^{-1}$ that keeps it within the quasi-static range to allow comparison with the analytical study qualitatively and with the physical experiments presented later quantitatively. To ensure that inertial effects are limited, the kinetic energy is checked within the analyses such that it does not exceed 5% of the internal energy.

### 3.1. Unit cells

**Figure 6.**
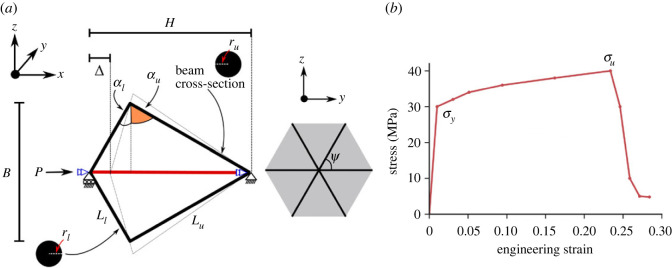
(*a*) Three-dimensional unit cell of the flint arrowhead geometry under compression showing annotated views in the *xz* and *yz* planes, respectively. (*b*) Predefined elasto-plastic material properties for Nylon showing stress versus engineering strain with yield stress *σ*_*y*_ and ultimate stress at failure *σ*_*u*_ taken from tests conducted in [35].

Preliminary studies are conducted on a three-dimensional flint arrowhead unit cell under compression, which is a three-dimensional extension of the planar arrowhead geometry considered in the analytical model. To model these unit cells, rigidly connected three-dimensional quadratic three-noded Timoshenko beam elements (B32) are used with circular solid sections with a Young’s modulus *E* = 2 GPa, which represents the material property of Nylon as that was selected for the tested physical specimens. Since inertial effects are considered, albeit minimally owing to the quasi-static nature of the present study, the material density of Nylon 1150 kg m^−3^ is used for all models. Four elements per structural member are used, since a mesh convergence study demonstrated that this was sufficient to capture expected deflected shapes [34]. The boundary and loading conditions are given in figure 6*a* with the right end-node pinned, constraining all translational displacements. A displacement-driven load is applied at the left end-node with the addition of a roller boundary allowing only displacement in the *x*-direction as shown. Rotation about the *x*-axis is also constrained for both the pinned and roller ends to eliminate twisting of the unit cell under compression, which in practice is unlikely to be a significant issue since the network of cells would make the lattice arrangement torsionally very stiff. The initial inclination angles and lengths are given in table 3. The unit cell model described in figure 6 can be related to some extent to the rigid link–spring model presented in figure 2. The rigid links obviously relate to the individual members of the cell, whereas the individual rotational springs at the nodes represent the rotational stiffnesses of the cell joints. The longitudinal spring linking points D to E in figure 2*a* relates to the axial stiffness of the middle strut, while the transverse spring linking points F to G in figure 2*a* relates to the bending stiffness of the middle strut to provide its buckling load and model its post-buckling stiffness.

**Table 3.** Initial lower and upper angles of the flint arrowhead unit cell *α*_*l*_ and *α*_*u*_, respectively, alongside length *B,* which is equivalent to lengths *H* and *L*_*u*_.

*α*_*l*_ (deg)	*α*_*u*_ (deg)	*B* (mm)
$15^{\circ }$	$60^{\circ }$	14

The horizontal end-shortening arising from the displacement-driven load on the left is given by Δ. As shown in table 4, for the cases labelled ‘FU’, four different cell cases with different lower member radii *r*_*l*_, which are 25%, 50%, 75% and 100% of the upper inclined member radii *r*_*u*_, are analysed such that a similar spectrum of results are studied as in the analytical study (figure 3); a constant relative density across all model cases is maintained by varying all radii values. The middle strut, shown in red in figure 6, is assumed to have the same cross-section radius (*r*_*m*_) as *r*_*l*_ and is therefore examined for the four different magnitudes relative to *r*_*u*_, as detailed above. The principal objective for these parametric changes is to determine their effects on the snap-through behaviour of a flint arrowhead and establish a favourable configuration that has an equilibrium response with a high load-carrying capacity and a near-zero post-buckling stiffness. Note also that a small half-sine wave imperfection of amplitude *H*/1000 is introduced to the middle strut to ensure it buckles, thus avoiding numerical convergence issues occurring when the instability is triggered.

**Table 4.** Element radii for the FE model cases: each is shown as a number pair with the first number representing *r*_*u*_ while the second number represents the equal *r*_*l*_ and *r*_*m*_ values. Four unit cell (FU) cases are studied where *r*_*l*_ = *r*_*m*_ that are 25%, 50%, 75% and 100% of *r*_*u*_; for the four stack (FS) cases these respective values are 40%, 45%, 50% and 75% of *r*_*u*_. The full panels FP (ungraded) and FP* (graded) have their *r*_*l*_ and *r*_*m*_ values at 45% of *r*_*u*_ values with *r*_*m*_ being reduced to 40% of *r*_*u*_ in the graded panel (FP*) for the central one-third region along the length.

cases	unit cell element upper, lower and middle element radii (mm): $\left\{r_{u},(r_{l}\;\&\;r_{m})\right\}$
FU (4)	{0.70, 0.18}; {0.66, 0.33}; {0.60, 0.45}; {0.55, 0.55}
FS (4)	{0.82, 0.32}; {0.82, 0.37}; {0.82, 0.41}; {0.82, 0.58}
FP (1)	{0.82, 0.37}
FP* (1)	outer one-thirds along length: {0.82, 0.37}
	central one-third: *r*_*u*_ = 0.82 mm, *r*_*l*_ = 0.37 mm and *r*_*m*_ = 0.32 mm

### 3.2. Flint arrowhead cellular stack

**Figure 7.**
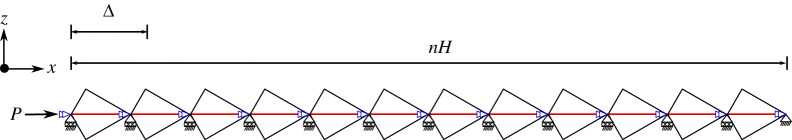
A schematic of 12 flint arrowhead cells in series under axial compression *P*. Note that the end of each cell is constrained vertically to inhibit global buckling.

In the analytical study presented in §2.2.2, a stack of five cells were studied to obtain qualitative information on how a stack of multiple cells would behave when loaded axially. For its purpose, a stack of five cells was sufficient in terms of obtaining all the equilibrium paths, understanding their provenance, while avoiding convergence issues that often occur when solving a large system of nonlinear algebraic equations numerically, especially where bifurcating instabilities abound. In the present FE study, labelled as case ‘FS’ in table 4, a longer stack of 12 flint arrowhead cells arranged in series, which represents a more realistic length within a lattice structure, is analysed to establish the unidirectional influence of the snap-through mechanism prior to analysing the full lattice geometry. As shown in figure 7, the arrowheads are orientated in the horizontal direction with a pinned constraint at the right end-node and a displacement-driven load *P* applied at the left end-node. The central nodes are placed on rollers allowing only horizontal displacement such that global buckling is inhibited since such an instability is unlikely within a stack that is part of a lattice structure; other boundary constraints described for the unit cell case also apply presently.

### 3.3. Lattice panel

**Figure 8.**
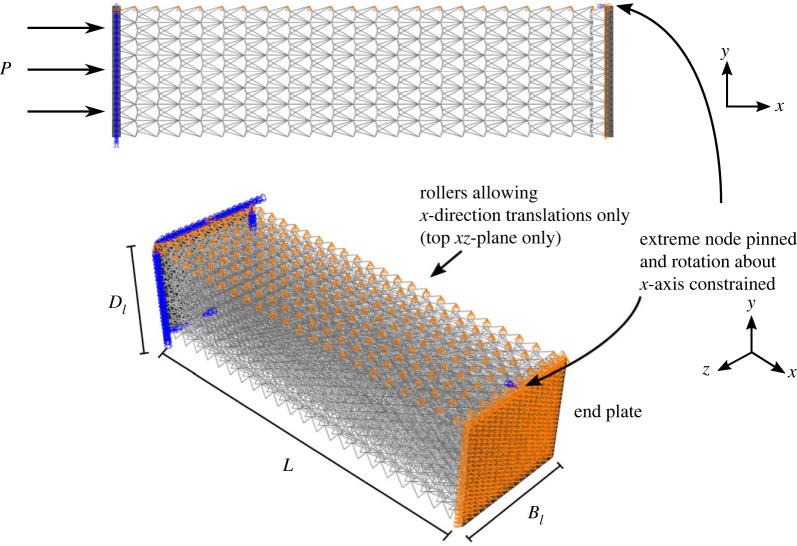
Full three-dimensional panel with flint arrowhead lattice core and roller boundary conditions constraining the *y* direction displacement on one edge. A monotonically increasing pressure load is applied to one of the end plates. Two full panels are studied, ‘FP’ with every cell being identical, and ‘FP*’ where the cells in the middle third along the lattice length have a reduced *r*_*m*_ value.

Of course, studying a single stack of cells naturally only provides a small set of the interactions between individual members and cells that can occur within an entire lattice. Hence, it is necessary to model full lattices, particularly if the FE models need to be compared with physical tests. Figure 8 shows an elevation and isometric view of a typical core lattice arrangement for the three-dimensional panel. The first and last rows within the core lattice are composed of the auxetic arrowhead configuration since this geometry allows for increased joint connections to a flat (end-plate) surface. The remainder of the volume is populated with the flint arrowhead geometry, i.e. a hexagonally tessellated stack of flint arrowhead cells. A monotonically increasing pressure load is applied to one end-plate to simulate a blunt impact-type loading condition (but without accounting for the high acceleration associated with impact loads). The edge nodes of the lattice on one side of the panel, as shown by the orange rollers in figure 8, constrain lateral movement. This provides structural integrity to the lattice such that the cells snap-through in one direction, without a Euler-type global buckling being triggered.

Two full lattice cases are studied (table 4): case ‘FP’ has uniform properties across the panel, whereas case ‘FP*’ has a controlled yet discrete variation of the geometrical properties of the lattice elements, referred to as *geometrical grading* to improve the mechanical response by engineering a controlled buckling sequence (as distinct from ‘functional grading’ which is generally used for a continuous variation in mechanical properties), as was achieved in the analytical model (figures 4 and 5). Numerical experimentation showed that only the middle struts of the flint arrowheads require variation since it was found that grading other members within the flint arrowhead cellular geometry produced regions that were excessively stiff and acted effectively as boundaries between gradation zones. This, in turn, halted the desired buckling propagation and triggered re-stiffening, usually attributed to densification [34]. However, the subtle changes applied to the middle struts are in accord with previous research [36–38], where only gradual changes in cell stiffness have been demonstrated to be desirable for structural isolation applications such that a smooth plateauing response is obtained.

### 3.4. Results for unit cells

**Figure 9.**
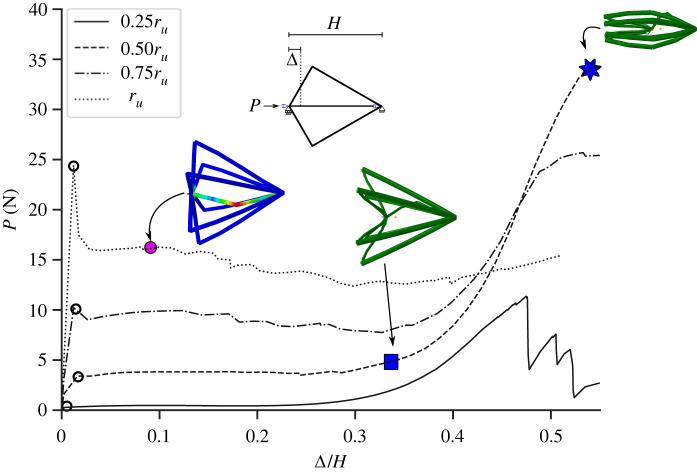
Mechanical responses of the unit cell cases ‘FU’ obtained from FE showing axial load *P* versus normalized axial end-shortening Δ/*H* for four different lower member radii at values of 25%, 50%, 75% and 100% of the upper member radii with cell deformations corresponding to labelled points in the load–displacement response. Note: in the cell deformation plots, the red regions signify the onset of material failure while dark blue represents low plastic strain.

The mechanical responses of a unit cell of the flint arrowhead geometry is examined with elasto-plastic material properties of Nylon adapted from an experimental study conducted by Villette *et al.* [35] on similarly sized three-dimensional-printed specimens. As shown by the stress–strain curve in figure 6*b*, a yield stress of 30 MPa is given with the ultimate stress at failure being 40 MPa. Owing to the snap-through behaviour of this geometry, four different cases with varying radii of the lower member (shown in table 4) are examined to analyse its parametric influence. The dimensions of the middle struts have been kept to be the same size as the lower member radii and altered accordingly with the upper members slightly varied to maintain a constant relative density of 0.2. Figure 9 shows the load *P* versus axial end-shortening Δ/*H* responses of these flint unit cells with the initial upper and lower inclination angles $\alpha_{u}=60^{\circ }$ and $\alpha_{l}=15^{\circ }$, respectively (figure 6 and table 3). The upper angle was fixed such that the cell was as tall as wide, while a preliminary sensitivity analysis examining varying lower member inclinations showed that the most favourable lower angle *α*_*l*_ was $15^{\circ }$ such that a controlled snap-through response caused only minimal plasticity to be generated at the joints. As expected, there is a significant increase in the initial load-carrying capacity between the model cases where *r*_*l*_ = 0.25*r*_*u*_ and cases where all the members are equal (*r*_*l*_ = *r*_*u*_). This can be attributed principally to the increase in axial and bending stiffness of the middle strut as its radius is increased. The middle strut is pivotal in the initial phase of the load–displacement response since it is under direct compression and therefore is the most effective in resisting the external load. However, since the lower members also govern the geometrical phase transition from conventional to auxetic behaviour of the unit cell, increasing these inclined member radii also increase their axial and bending stiffnesses along with their respective orientations, which in turn stiffens the unit cell overall.

Although increasing the radii of the lower members and middle strut can increase its load-carrying capacity, overly stiffening these members can have an adverse effect for the post-buckling response, as shown in figure 9 by a drop in the capacity beyond the critical buckling load of the case where *r*_*l*_ = *r*_*u*_. Figure 9 shows the equivalent plastic strain of that unit cell through coloured contours on the deformed cell images, whereby, for the chosen Nylon material a value above 0.2 indicates the onset of fracture. This material failure is attributed to be the cause of the sudden drop in the capacity and signifies the importance of the middle strut even beyond the critical buckling point acting to enable a more gradual transition from the conventional to the auxetic configuration. Despite having a lower overall capacity, the case where *r*_*l*_ = 0.5*r*_*u*_ shows the best post-buckling response out of the four cases. Although the analytical model presented earlier guided how the cell deforms in terms of snap-through buckling, the fidelity of the FE models provides more detailed practical information for how the deformed cells are further affected by local material damage.

### 3.5. Results for flint arrowhead stacks

**Figure 10.**
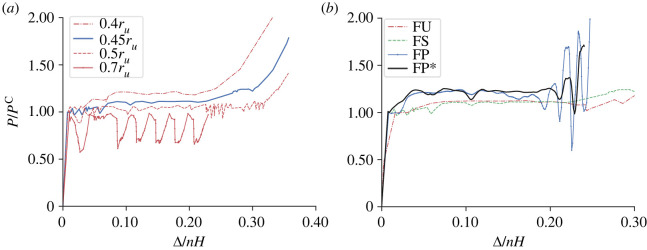
Normalized load versus normalized axial end-shortening responses for four variations of the flint arrowhead stack under compression: (*a*) single stack with various lower member and middle strut radii (*r*_*l*_ and *r*_*m*_, respectively); (*b*) comparisons for the following cases: unit cell (FU), a single stack (FS), a uniform lattice panel (FP) and a graded lattice panel (FP*).

Similar to the unit cell study, the behaviour of several flint arrowhead stacks are examined presently. The lower member and middle strut radii are varied to establish the influence that these parameters have on the overall mechanical response and, by implication, on the energy absorption and structural isolation characteristics. Figure 10*a* shows the normalized load *P*/*P*^C^ versus normalized axial end-shortening Δ/*nH* of four flint arrowhead stacks, those with relative lower member radii *r*_*l*_/*r*_*u*_ being 40%, 45%, 50% and 70%.

There are some differences observed in the initial gradient representing the axial stiffness of the considered stacks, whereby the case with *r*_*l*_/*r*_*u*_ = 0.4 shows a reduced stiffness in comparison with the remainder. However, in general, these initial differences which, as established from the unit cell model, are governed by the stiffness of the middle strut(s), are small. By contrast, significant differences are observed in the post-buckling response. For cases with smaller lower member radii, such as the case with *r*_*l*_ = 0.4*r*_*u*_, a re-stiffening response as well as an earlier onset of densification is observed. This is predominantly owing to the reduced stiffness of the lower members allowing an easier geometrical phase transition and subsequent cell densification. As the lower members are thickened, as in the cases where *r*_*l*_/*r*_*u*_ are 0.45 and 0.5, there is more resistance to the transition, leading to greater plateauing whose paths are more uniform in terms of load, which are ideal responses for structural isolation. Indeed, there appears to be a threshold for this desired outcome, which in this case is when the lower members and the middle struts are set to approximately 0.45*r*_*u*_. For this case, shown by the solid line curve in figure 10*a*, there are minimal disruptions in the post-buckling range as the cells sequentially buckle and there is a significant extent of the desired quasi-zero stiffness prior to densification. However, as the lower members are thickened further, the mechanical response exhibits less smooth behaviour with disruptive localized deformation patterns in the case where *r*_*l*_/*r*_*u*_ = 0.5. For the stiffest case (*r*_*l*_/*r*_*u*_ = 0.7), the cells effectively begin to act independently of each other; an initially negative stiffness is exhibited as an individual cell buckles that subsequently densifies in a similar way to the cases presented in figure 3*a*, particularly where the middle strut lateral spring stiffness ($k_{b_{1}}$) is relatively large. Moreover, artificially stiff boundaries are created between cells adjacent to those densifying that leads to the large oscillations in the load once those adjacent cells also buckle. Of course, as surrounding cells are added to these single stacks to form complete lattices, the sensitivities to the differences in thickness between the upper, lower and middle struts would also vary, but this set of results provides clear evidence that tuning geometries at an individual cellular level can affect the overall response significantly.

### 3.6. Results for entire lattices

An entire lattice of the flint arrowhead geometry is sandwiched between the two end-plates as a core component of a panel and is analysed. Figure 10*b* shows a comparison of the normalized load *P*/*P*^C^ versus the normalized axial end-shortening Δ/*nH* of the different scale models, starting from the unit cell ‘FU’ with *n* = 1, the flint stack ‘FS’ with *n* = 12, and the final two iterations of the entire flint panel ‘FP’ and ‘FP*’. In the latter case (FP*), the panel has been geometrically graded such that the middle struts in the central region of the lattice have a reduced radius. The responses of the unit cell and stack are practically identical, both with the desired high initial stiffnesses and near-zero post-buckling stiffnesses. The panels have similar characteristics, with the main difference being the more gradual transition from the initial stiffness to the more plateauing post-buckling response. In fact, in the FP case, there is an initial plateauing beyond the critical buckling point, which is attributed to the softening of the lattice as the middle struts initially buckle, and then a small re-stiffening path before the largely plateauing response as successive cells gradually transition between the conventional to auxetic phases. The post-buckling response also shows regions with disturbed and undulating paths caused by the reduced stiffness of the overall lattice as hints of global, Euler-type, buckling appear to be triggered. However, these minimal disturbances in the post-buckling path are eliminated through geometrical grading in the FP* panel, as well as the reduction in the large oscillations observed at high Δ/*nH* values as the densification phase takes hold.

## 4. Physical experimentation

**Figure 11.**
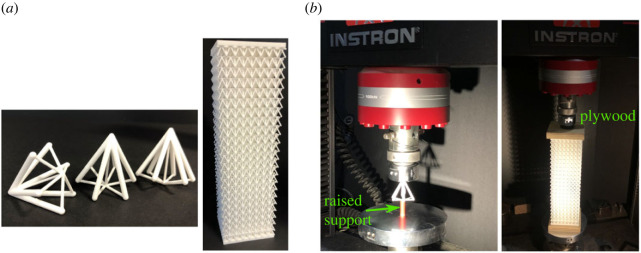
(*a*) Three-dimensional-printed models for the unit cell (as-printed cell height: *H* = 37.8 mm) and panel (8 × 8 × 23 cells, as-printed panel length *L* = 293.4 mm) and (*b*) experimental set-up for the compression test rig used for the unit cell and panel, respectively.

To validate the FE model, a series of unit cell and lattice prototypes were designed and produced through additive manufacturing for physical testing. The three-dimensional design of both the unit cell and panels was conducted within the commercial parametric design software Rhinoceros 3D [39] (Rhino) aided by Grasshopper, a graphical algorithm editor and a plug-in within Rhino. Table 5 provides the different radii values for the unit cell and panel specimens. A relative density of 0.2 was maintained for all the designs, which remains well within the domain of acceptable relative densities for cellular materials [22]. Samples of each of the unit cell and the panel models, as depicted in figure 11*a*, were fabricated using the industrial three-dimensional printer Formiga P 110 Velocis [40]. This three-dimensional printer uses a high precision laser spot with a small focus diameter to print intricate structures through the selective laser sintering (SLS) method. In the SLS printing technique utilizing thermoplastic materials, the structure is merely supported by excess powder material remaining from the sintering process. Therefore, simple dusting and removal of this powder material is the only post-processing required. The material used is Polyamide (PA 2200), commonly known as Nylon, chosen for its desirable material properties and production quality; for the particular three-dimensional printer used, the printed material has an average Young’s modulus of 1.7 GPa and the typical density for laser-sintered parts is 930 kg m^−3^, both values that are taken from the manufacturer’s data sheet [41].

**Table 5.** Radii dimensions for the upper *r*_*u*_, lower *r*_*l*_ and middle *r*_*m*_ struts for the unit cell and panel models; $r_{m}^{*}$ is the radius of the middle struts in the central region of the panel, which have been reduced to initiate buckling in that region first; *L*_*u*_ is the length of the upper members for each case. Note that cell angles $\alpha_{u}=60^{\circ }$ and $\alpha_{l}=15^{\circ }$ are used in all cases.

cases	*r*_*u*_ (mm)	*r*_*l*_ (mm)	*r*_*m*_ (mm)	$r_{m}^{*}\;(\mathrm{mm})$	*L*_*u*_ (mm)
unit cell	1.62	0.81	0.73	n.a.	37.8
panel	0.92	0.46	0.46	0.44	12.6

**Figure 12.**
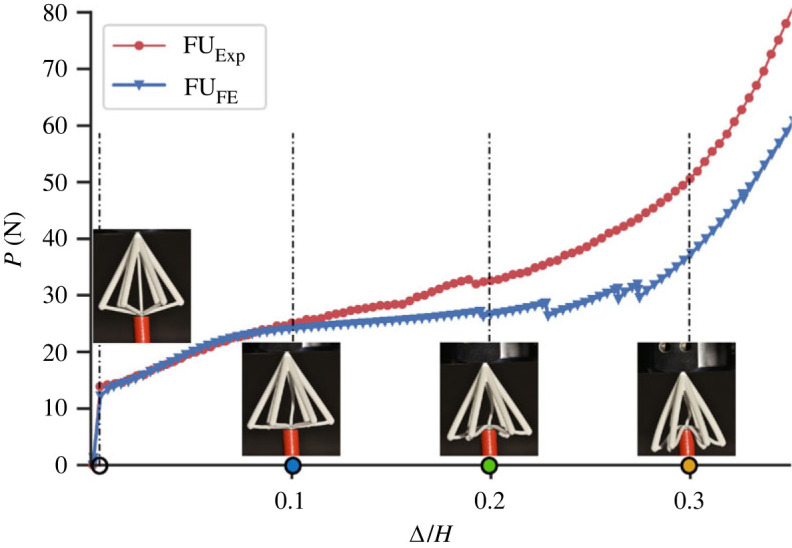
Load *P* versus normalized axial end-shortening Δ/*H* responses comparing the experimental (FU_Exp_) and FE (FU_FE_) unit cell models with corresponding deformations from the experimental test. Note that the lower support has a finite width that affects the behaviour once the cell becomes auxetic and the lower members make contact with the support surface.

Physical tests were conducted on a flint arrowhead unit cell as well as on a panel with a lattice core comprising the flint arrowhead geometry, to verify the responses of the numerical results. Figure 12 shows the load *P* versus normalized axial end-shortening Δ/*H* response for the flint arrowhead unit cell case comparing the experiment with the FE model. The displaced unit cell from the experiment is shown in figure 12, at four distinct locations corresponding to the load–displacement response. There is an excellent match between the experiment and the FE model, particularly where Δ/*H* < 0.1. The general response, in both cases, can be subdivided into the familiar three stages: the pre-buckling axial compression stage shown by the initially steep linear part of the response; followed by the post-buckling path with an initial sharp drop in stiffness that then reduces further, and lastly the densification phase where the stiffness resumes increasing. Beyond the range where Δ/*H* > 0.1 the experimental result begins to diverge from the more plateauing response observed in the FE model, as supported by the corresponding test photographs in figure 12; this can be attributed to the lower support, which is modelled numerically as a point but in reality has a finite width that comes into contact with the lower members snapping through and acts artificially to increase the overall stiffness.

According to the deformations presented in figure 12, the middle strut is the first member within the unit cell to buckle and occurs at a normalized displacement just beyond that marked by the hollow circle. Owing to the unit cell initially having a conventional geometry, lateral expansion is observed before the snap-through response of the lower members, see the deformation marked by the blue circle, where Δ/*H* = 0.1. It is also worth noting that only the middle strut has buckled at this stage with minimal bending observed in the other members. As the geometry transitions to an auxetic phase (observed in the deformations marked by the green circle, where Δ/*H* = 0.2), the unit cell begins to contract laterally. This response eventually leads to a secondary rise in the load because the lower members are under tension while the thicker upper members begin to bend, shown by the curved upper members in the deformations marked by the brown circle, where Δ/*H* = 0.3.

**Figure 13.**
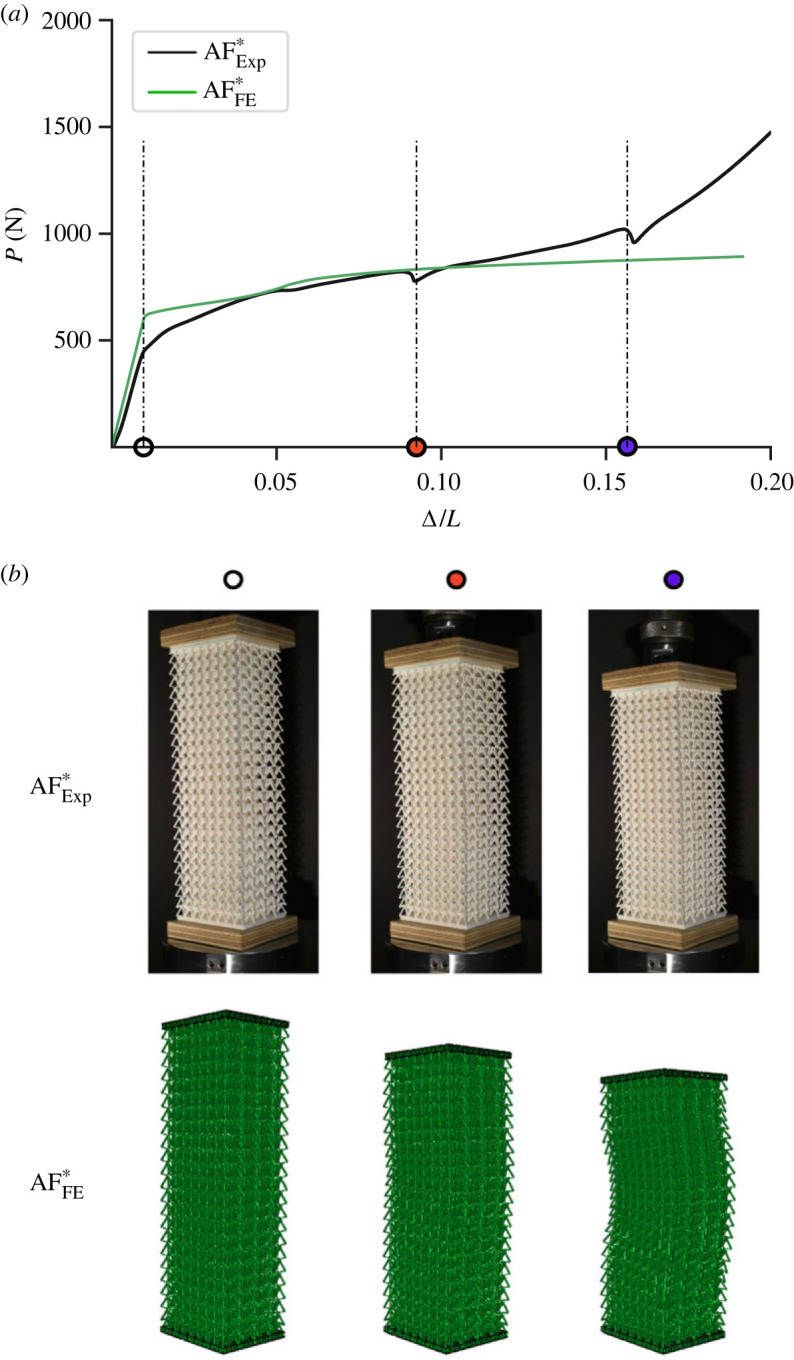
(*a*) Load *P* versus normalized axial end-shortening responses for the experimental ($AF_{\mathrm{Exp}}^{*}$) and FE ($AF_{\mathrm{FE}}^{*}$) panels with (*b*) showing corresponding deformations at three distinct locations.

Cases comparing the experimental and FE model for the flint arrowhead panel, $AF_{\mathrm{Exp}}^{*}$ and $AF_{\mathrm{FE}}^{*}$, respectively, are shown next. The asterisks indicate that the central region of these panels contains a region with reduced middle strut radii (*r*_*m*_), in an identical fashion to the FE case FP* (table 4). Unlike the model in §3.6, the printed panel is scaled down by 10% to allow the overall structure to fit within the printable volume, with an equivalent FE model also created, hence the new naming convention. As shown in figure 13*a*, a very good comparison is seen in the mechanical responses. Small discrepancies are observed in the early stages near the critical buckling load mark, indicated by the hollow circle. The experimental result shows a more gradually changing slope as the middle struts buckle and the cells transition from conventional to auxetic forms. This could be owing to differences in the joint stiffnesses between the actual joint rotational stiffness of the lattice cells in the three-dimensional-printed specimen being slightly more flexible compared with the more idealized representation in the FE model. The other point of divergence between the experiment and the FE model is in the latter stages of the response. An earlier rise in stiffness is seen in the experiment where Δ/*L* > 0.15, which may be attributed to more extreme deformations in some individual cells causing an earlier onset of densification. By contrast, the FE model appears to display signs of global buckling at that stage, as shown by the corresponding deformations in figure 13*b*, which would also tend to reduce the system stiffness further. The tests were conducted to complete panel failure, which of course goes well beyond the scope of the analytical modelling; please refer to the electronic supplementary material for the full results, where experimental evidence of a small extent of global buckling is also highlighted.

## 5. Discussion

The principal objective of the present work is to enhance energy absorption and structural isolation performance of a protective layer from impact loads by harnessing the stiffness changing characteristics from elastic buckling within cellular structures that form a mechanical metamaterial. Currently, the flint arrowhead geometry, both as a unit cell as well as within larger stacks and an entire lattice, has been investigated analytically through a simplified model comprising rigid links and springs, numerically using commercial FE software to model physically realistic joint and element conditions, and through physical testing. Fundamentally, the analytical models were able to highlight the key features of the model that provide the desired mechanical response for the aforementioned application, which essentially transforms the behaviour from an initially high stiffness phase that generates a significant load-carrying capacity to a low (quasi-zero) stiffness phase that inhibits stress propagation while, theoretically at least, remaining elastic. In combination, this leads to excellent energy absorption and structural isolation characteristics that can shield a vulnerable structure that needs protection from the applied load. At the unit cell level, inclusion of a middle strut showed significant advantages for increasing the buckling load while the joint rotational springs, largely responsible for the intricacies of the snap-through response, dictated the overall post-buckling stiffness. The increased load-carrying capacity enhances the case for using such cellular structures since much greater quantities of energy can be absorbed during the buckling of each cell. Moreover, the advantage of being able to predict and tune the post-buckling stiffness by adjusting the various spring stiffnesses provides the potential for optimizing the lattice structure for different loading cases.

The findings from the FE study essentially concurred with those from the analytical model for the unit cell, where similar stiffness variations were initiated through changes to member radii, which affects both the axial and bending stiffness of the individual members of the cell structure. The middle strut radius was modified to change the initial stiffness as well as the intrinsic load-carrying capacity of the cell and, in this instance, the radii of the lower members were also modified to tune the snap-through buckling response. The most desired response with the presently selected materials and arrangement was shown to occur when the lower members and middle strut radii were set to 50% of the upper member radii. Increasing the stiffness beyond this threshold led to a distinctly unstable post-buckling response with a loss in load-carrying capacity and the potential to damage the material prematurely.

The analytical model was also enhanced to join several flint arrowhead cells together as a stack. Under loading, it was shown to produce a desirable sequential buckling response, the benefits of which are that it can extend the desirable quasi-zero stiffness response range. This potentially allows the lattice to absorb much more energy while protecting the aforementioned vulnerable structure and effectively makes the system scalable with the energy absorbency being related to the number of cells that buckle sequentially within the stack. The more physically realistic FE models of the flint arrowhead stack were then used to investigate methods of tuning the post-buckling response. Similar to the corresponding unit cell study, for the selected materials and arrangement, the lower members and middle struts were found to work best when their radii lay within the range 45–50% of the upper member radii. Below this range, a re-stiffening post-buckling response was observed that violated the quasi-zero stiffness requirement and anything above the specified range showed much more unstable oscillations in the mechanical response. These oscillations were attributed to local cells densifying before triggering instability in adjacent cells, both of which are not ideal particularly for structural isolation applications. The desired response achieved through these parametric choices were also scalable since largely similar responses were observed for full panels with flint arrowhead lattice cores. The post-buckling response could also be further tuned through modifications to middle strut radii at particular locations throughout the span of the stack. Finally, experimental tests were conducted both on a unit cell and a lattice panel, both of which were in very good agreement with the FE model. The pre- and post-buckling paths for both cases matched, with slight discrepancies only emerging from the experimental set-up or imperfections in the three-dimensional-printed specimens. The experimental work not only verifies the numerical models but highlights the practicality of the work where a system may be developed further for use in practice. Moreover, since the majority of the responses observed currently are within the elastic range, these promising results open up the scope for further work that could examine full recoverability of such lattices that may be called into service in quick succession. Moreover, protection systems that are presently single-use may be able to be sensibly repaired and hence re-used with the advantages that has in terms of resource use and sustainability.

## 6. Concluding remarks

The nonlinear mechanical behaviour of a mechanical metamaterial composed from a cellular flint arrowhead geometry was examined through a bottom-up approach of initially investigating the response at a unit cell level and later within a stack of cells and then an entire lattice core. Theoretical, numerical and experimental methodologies were used to understand the controlling mechanisms. The effects of varying different stiffnesses by adjusting geometric properties and arrangements were used to tune the mechanical response to give the desired response for enhanced energy absorbency and structural isolation. The present work provides an analytical framework for engineering and potentially optimizing such lattices so that elastic buckling may (at least partially) replace material damage as the principal mechanism for absorbing energy while protecting a vulnerable structure and remaining undamaged. This potentially opens up the opportunity to devise a repairable and sustainable protection system that has the advantages of not being single-use and hence not requiring immediate replacement once mobilized into service. This could, in turn, reduce whole-life costs while increasing structural resilience such that critical infrastructure is not left unprotected whereas current practice would necessitate replacement of the protective system.

## Data Availability

All relevant data are available via the following link: https://tinyurl.com/MetaLattice. The data are provided in electronic supplementary material [42].
